# The Influence of Coronary Artery Disease in the Development of Aortic Stenosis and the Importance of the Albumin Redox State

**DOI:** 10.3390/antiox11020317

**Published:** 2022-02-05

**Authors:** Tamara Sastre-Oliva, Nerea Corbacho-Alonso, Diego Albo-Escalona, Juan A. Lopez, Luis F. Lopez-Almodovar, Jesús Vázquez, Luis R. Padial, Laura Mourino-Alvarez, Maria G. Barderas

**Affiliations:** 1 Department of Vascular Physiopathology, Hospital Nacional de Paraplejicos, Servicio de Salud de Castilla-La Mancha (SESCAM), 45071 Toledo, Spain; tsastre@sescam.jccm.es (T.S.-O.); ncorbacho@sescam.jccm.es (N.C.-A.); dmartinalbo@externas.sescam.jccm.es (D.A.-E.); 2 Cardiovascular Proteomics Laboratory and Centro de Investigación Biomédica en Red Enfermedades Cardiovasculares (CIBER-CV), Centro Nacional de Investigaciones Cardiovasculares (CNIC), 28029 Madrid, Spain; jalopez@cnic.es (J.A.L.); jesus.vazquez@cnic.es (J.V.); 3 Cardiac Surgery, Hospital Virgen de la Salud, Servicio de Salud de Castilla-La Mancha (SESCAM), 45004 Toledo, Spain; lopezalmodovar@yahoo.es; 4 Department of cardiology, Hospital Virgen de la Salud, Servicio de Salud de Castilla-La Mancha (SESCAM), 45004 Toledo, Spain; lrodriguez@sescam.org

**Keywords:** coronary artery disease, calcific aortic valve disease, aortic valve stenosis, redox state, cysteine oxidation

## Abstract

Calcific aortic valve and coronary artery diseases are related cardiovascular pathologies in which common processes lead to the calcification of the corresponding affected tissue. Among the mechanisms involved in calcification, the oxidative stress that drives the oxidation of sulfur-containing amino acids such ascysteines is of particular interest. However, there are important differences between calcific aortic valve disease and coronary artery disease, particularly in terms of the reactive oxygen substances and enzymes involved. To evaluate what effect coronary artery disease has on aortic valves, we analyzed valve tissue from patients with severe calcific aortic stenosis with and without coronary artery disease. Proteins and peptides with oxidized cysteines sites were quantified, leading to the identification of 16 proteins with different levels of expression between the two conditions studied, as well as differences in the redox state of the tissue. We also identified two specific sites of cysteine oxidation in albumin that have not been described previously. These results provide evidence that coronary artery disease affects valve calcification, modifying the molecular profile of aortic valve tissue. In addition, the redox proteome is also altered when these conditions coincide, notably affecting human serum albumin.

## 1. Introduction

Calcific aortic valve disease (CAVD) or aortic valve (AV) stenosis is a progressive condition in which a narrowing of the AV area occurs due to a thickening and calcification of the leaflets, the progression of which is associated with worse outcomes [1,2,3]. AV degeneration shows several similarities with coronary atherosclerosis, even sharing many clinical risk factors [4]. In fact, the presence of coronary artery disease (CAD) is a negative predictive indicator in patients with CAVD [5,6,7] and hence it is recommended that coronary arteries are evaluated by invasive coronary angiography or coronary computed tomography (CT) angiography prior to the prescription of surgical or transcatheter AV replacement [2].

The processes of degeneration and calcification in CAVD and CAD have similar onsets. Both pathologies initiate through endothelial dysfunction, which favors the infiltration of inflammatory cells and lipid deposition in the tissues [8,9,10]. However, the AV has many fewer smooth muscle cells and it undergoes more severe calcification [11]. Furthermore, AVs and arteries are functionally distinct structures, such that tissue stiffness has different consequences and the most severe clinical manifestations have different causes. The clinical manifestations in CAVD are due to obstructed blood flow while in CAD, plaque stability is key to avoid the release of prothrombotic agents [12,13]. Importantly, one mechanism that contributes to the calcification of vascular tissues is oxidative stress [14,15,16]. This happens when there is an imbalance between the production of reactive oxygen species (ROS) and the ability of the biological system to eliminate those radicals or repair the damage they provoke. These free radicals react with nearby molecules, including the proteins with sulfur-containing amino acids (e.g., cysteines, Cys) that are the most prone to oxidation [17]. This alters the structure and function of these proteins, their interactions with other proteins or their turnover rates. Significantly, important differences between CAVD and CAD involve ROS and certain enzymes [18,19].

The association between CAVD and CAD has been the focus of some studies, including those based on the moderate-to-large cohorts of the Progression of AtheRoscleroticPlAqueDetermIned by Computed TomoGraphic Angiography IMaging (PARADIGM) registry or the Multi-Ethnic Study of Atherosclerosis (MESA) [20,21]. Nevertheless, these works are prospective studies centered on different clinical parameters. Here, we performed, for the first time, the analysis of the AV directly to evaluate differences at the tissue level and to assess the effect of CAD on the mechanisms of AV calcification, as our hypothesis is that the presence of CAD may modify the course of CAVD. We used a filter-aided stable isotope labeling of oxidized Cys (FASILOX) approach, a novel multiplexed proteomic strategy that allows proteins and oxidized Cys (oxCys) sites to be quantified simultaneously [22]. We not only found different protein profiles in the two CAVD groups studied, with or without CAD, but also, differences in the redox status of the tissue despite their high degree of calcification. Moreover, we identified two specific sites of cysteine oxidation in albumin that have not previously been described that may be important in these events.

## 2. Materials and Methods

### 2.1. Patient Selection

**Figure 1:**
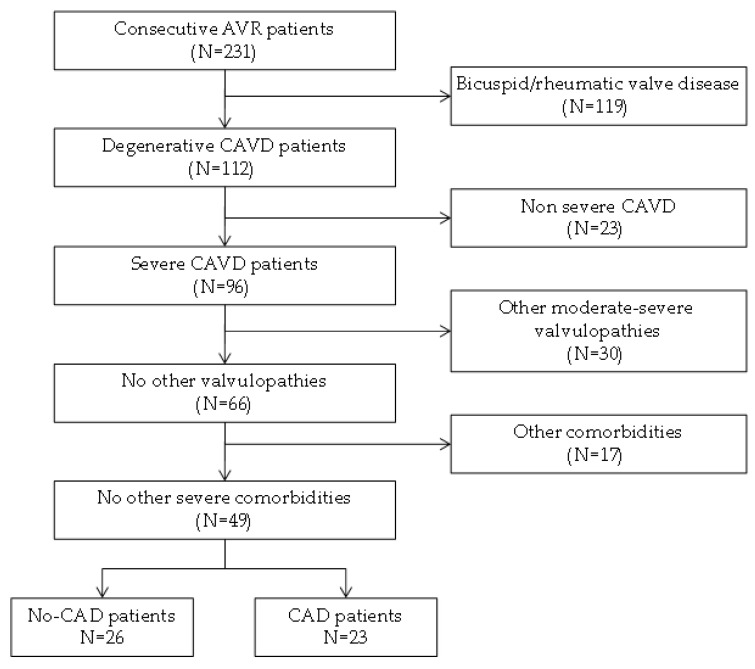
Flowchart of patient recruitment and inclusion. In total, 231 consecutive patients who were diagnosed with aortic stenosis and recommended for surgery were selected. After the application of exclusion limits, 26 and 23 patients without and with coronary artery disease remained. Finally, 22 patients of each group were selected to avoid significant differences between groups. CAVD, calcific aortic valve disease; CAD, coronary artery disease.

Heart valves and peripheral blood samples were obtained from consecutive patients with severe CAVD who underwent AV replacement at the Hospital Virgen de la Salud (Toledo, Spain) (Figure 1). 

Patients with any severe morbidity (ischemic heart disease with ventricular dysfunction, and end-stage chronic kidney disease), bicuspid AV, a family or personal history of aortopathy, rheumatic valve disease and moderate or severe mitral valve disease were excluded from the study. After selection, participants were classified into two study groups according to the presence/absence of CAD and selected to avoid significant differences in terms of other important cardiovascular risk factors: age, gender, obesity, hypertension, dyslipidemia, and diabetes (Table 1). The AVs that were removed from the patients were washed in PBS, and immediately frozen and stored at −80 °C until the proteomics studies were performed. The blood samples were collected in tubes containing EDTA, centrifuged at 1125 g for 15 min and the resulting supernatant was immediately frozen at −80 °C until analysis.

This study was carried out in accordance with the recommendations of the Helsinki Declaration and it was approved by the ethics committee at the Hospital “Virgen de la Salud” (Toledo, Spain). Signed informed consent was obtained from all individuals prior to their inclusion in the study.

### 2.2. Proteomics Overview

The experimental strategy was carried out in three steps: (1) tissue sample preparation; (2) a discovery phase performed on 20 AVs using FASILOX [22,23] to quantify proteins and reversible Cys modifications simultaneously in the same experiment; (3) a verification phase in which targeted proteomics using parallel reaction monitoring (PRM) was carried out, along with Western blotting to analyze the total proteins and Cys oxidation.

#### 2.2.1. Tissue Sample Preparation and Isobaric Labeling

Protein extracts were prepared from homogenized tissue using ceramic beads (MagNaLyser Green Beads apparatus) in extraction buffer (50 mM Tris-HCl, 2% SDS at pH 8.5) supplemented with 50 mM iodoacetamide for the alkylation of Cys residues. The samples were boiled for 5 min and then incubated in the dark for 20 min at room temperature (RT). After centrifugation, the supernatant was collected and the extraction procedure repeated. The protein recovered was quantified and 200 µg of protein was digested following the protocol described previously [23]. The samples were then diluted with 7 M urea in 0.1 M Tris-HCl [pH 8.5] (UA buffer), and loaded onto 30 kDa centrifugal filter devices (NanoSep 30k Omega: Pall Life Sciences, NY, USA). Following a buffer exchange with UA buffer, the proteins were reduced in 10 mM DTT for 1 h, washed with 50 mM Hepes buffer and alkylated for 45 min in the dark using 20 mM methyl methanethiosulfonate (MMTS: Pierce, MA, USA) in UA. Excess alkylating reagent was eliminated by washing three times with UA buffer and three additional times with 50 mM ammonium bicarbonate. The proteins were digested overnight at 37 °C with modified trypsin (Promega, WI, USA) in 50 mM ammonium bicarbonate at a 40:1 protein:trypsin (*w*/*w*) ratio. The resulting peptides were eluted twice by centrifugation with 50 mM ammonium bicarbonate and 0.5 M sodium chloride. Trifluoroacetic acid (TFA) was then added to a final concentration of 1%, and the peptides were desalted on C18 Oasis-HLB cartridges and dried for further analysis. For stable isobaric labeling, the tryptic peptides were dissolved in 100 mM triethylammonium bicarbonate buffer and the peptide concentration was determined by measuring the amide bonds with the Direct Detect system (Millipore, MA, USA). Equal amounts of each peptide sample were then labeled using the 10-plex TMT Reagents (Thermo Fisher Scientific, MA, USA) previously reconstituted in 70 μL of acetonitrile (ACN). After incubation at RT for 2 h, the reaction was stopped over 15 min with 1% TFA and the peptides were combined. Samples were concentrated in a Speed Vac, desalted on C18 Oasis-HLB cartridges and dried for further analysis. To increase the proteome coverage, TMT-labeled samples were fractionated by high pH reversed-phase chromatography (High pH Reversed-Phase Peptide Fractionation Kit: Pierce) and concentrated as before.

#### 2.2.2. Protein Identification and Quantification

Labeled peptides were analyzed by LC–MS/MS using a C-18 reversed-phase nano-column (75 µm I.D. × 50 cm, 2 µm particle size, Acclaim PepMap RSLC, 100 C18: Thermo Fisher Scientific) in a continuous ACN gradient consisting of 0–30% B for 360 min and 50–90% B for 3 min (A= 0.1% formic acid, FA; B = 90% acetonitrile, 0.1% FA). A flow rate of 200 nL/min was used to elute the peptides from the nano-column to an emitter nanospray needle for real-time ionization and peptide fragmentation on a QExactive HF mass spectrometer (Thermo Fisher Scientific). The enhanced FT-resolution spectrum (resolution = 60,000) and the MS/MS spectra from the 15th most intense parental ions were analyzed in the chromatographic run, with dynamic exclusion set at 40 s. For peptide identification, all spectra were analyzed with Proteome Discoverer (version 2.1.0.81) using SEQUEST-HT (both from Thermo Fisher Scientific). A database search of the Uniprot database was performed, containing all sequences from human and contaminants (November, 2016; 70,902 entries), using the following search parameters: trypsin digestion with 2 maximum missed cleavage sites; precursor and fragment mass tolerances of 2 Da and 0.03 Da, respectively; TMT modifications at N-terminal and lysine residues as fixed modifications; and methionine oxidation, carbamidomethyl Cys and modifiedCys as dynamic modifications. Peptide identification was performed using the probability ratio method [24], and the false discovery rate (FDR) was calculated using inverted databases and the refined method [25], with additional filtering for a precursor mass tolerance of 15 ppm [26]. The peptides identified had an FDR ≤ 1% and only these peptides were used to quantify the relative abundance of each protein from reporter ion intensities. In this model, protein and peptide log2 ratios are expressed as standardized variables, i.e., in units of standard deviation according to their estimated variances: Zp values for peptide quantification, corrected for changes in protein abundance, and Zq values for protein quantification. Details of the statistical model or algorithm used have been described elsewhere [27]. 

#### 2.2.3. Verification: Targeted Proteomics

For the PRM analyses, 250 µg of each plasma sample was reduced with 50 mM DTT in 4% SDS, 100 mM Tris/HCl [pH 7.6] buffer for five minutes at 95 °C, and the samples were then diluted to 0.1% SDS with 8M urea and alkylated with 50 mM IAA. The samples were then cleaned with 100 mM ammonium bicarbonate and digested at 37 °C overnight with Trypsin/LysC (Mass Spec Grade: Promega Cat# V5078) at a ratio of 1:50 enzyme:protein following the FASP protocol [28]. The digestion was stopped with 1% TFA and the samples were evaporated to dryness in a SpeedVac, reconstituted in 100 µL of an aqueous solution of 3% ACN/1% FA and diluted 1/20 to a concentration of 125 ng/µLfor MS analysis.

The peptides were analyzed using an Orbitrap Fusion Lumos™ Tribrid mass spectrometer (Thermo Scientific), equipped with a Dionex Ultimate 3000 ultrahigh pressure chromatographic system (Thermo Scientific) and a TriVersaNanoMate as the nanospray interface (Advion Inc. Biosciences). Firstly, peptide mixtures were loaded onto a µ-Precolumn (300 µm i.d. × 5 mm, C18 PepMap100, 5 µm, 100 Å, C18 Trap column: Thermo Fisher Scientific) at a flow rate of 15 µL/min, and they were separated using a C18 analytical column (NanoEase MZ HSS T3 column, 75 µm × 250 mm, 1.8 µm, 100 Å: Waters) at a flow rate of 250 nl/min over a 150 min run in three consecutive steps using linear gradients (3–35% B for 120 min, 35–50% B for 5 min and 50–85 % B over 2 min), followed by isocratic elution in 85 % B for 5 min and stabilization to the initial conditions (A = 0.1% FA in water, B = 0.1% FA in ACN). The mass spectrometer was operated in a PRM or targeted mode. Targeted ions were isolated in the quadrupole with an isolation window of 1.6 and fragmented in the HCD cell with 28% collision energy. Fragment ions were detected in the orbitrap with 30k resolution. The scan range was set to 150–2000 *m*/*z*, the AGC target was 5 × 10^4^ and the maximum injection time was 54 ms. To confirm the targeted ions detected, MS1 was also acquired in the orbitrap with the resolution set to 120,000 (defined at 200 *m*/*z*) and a maximum injection time of 50 ms.The spray voltage in the NanoMate source was set to 1.70 kV. The RF Lens were tuned to 30% and the mass spectrometer was working in positive polarity mode.

We performed a database search with Thermo Proteome Discoverer v2.4.1.15 (PD) with Sequest HT as the search engine to build a spectral library in Skyline software. The databases used in the search were SwissProt Human (released 2020_06). We ran the search against targeted and decoy databases to determine the FDR. The search parameters included trypsin enzyme specificity, allowing for two missed cleavage sites, oxidation at M and acetylation at the protein N terminus as dynamic modifications. Peptide mass tolerance was 10 ppm and the MS/MS tolerance was 0.02 Da. Quantitative targeted MS/MS analysis was performed using Skyline v20.1.0.155 open source software [29]. A spectral library was generated in Skyline from database searches of the targeted MS/MS raw files with Proteome Discoverer v2.4.1.15 (Thermo), importing the final peptides selected manually within Skyline. Peaks were picked in an automated fashion using the default Skyline peak picking model, with Savitzky–Golay smoothing. Peak area integration was based on extracted ion chromatograms (XICs) of MS/MS fragment ions masses, typically y- and b-ions, matching specific peptides present in the spectral library. All transitions and peak area assignments were validated manually, and the list of monitored peptides are shown in Table 2.

#### 2.2.4. Verification: Western Blotting

Plasma samples were obtained from an independent cohort of patients with severe CAVD, with and without CAD. Equal amounts of protein (25 µg) from each patient were resolved by 8–10% SDS–polyacrylamide gel electrophoresis (SDS-PAGE) in a Bio-Rad Miniprotean II electrophoresis cell run at a constant current of 25 mA/gel. After electrophoresis, the proteins were transferred to a nitrocellulose membrane under a constant voltage of 20 V for 75 min and the membranes were stained with Ponceau S to guarantee an equal amount of protein was loaded for each patient. To analyze protein oxidation, prior to SDS-PAGE the samples were labeled with -*SulfoBiotics*- PEG-PCMal(Dojindo Molecular Technologies Inc., SB20-01, MD, USA), a 5 kDa Protein-SHifter, in accordance with the manufacturer’s instructions. Subsequently, the gels were exposed to UV light on a transilluminator to remove the Protein-SHifter and transferred using the usual protocol. The number of the reduced Cys residues was determined based on the molecular mass shift of the bands after electrophoresis. The membranes were then blocked for 1 h with 7.5% non-fat dry milk diluted in Phosphate-Buffered Saline-0.5%Tween 20 (PBS-T) and probed overnight with the primary antibody diluted in PBS-T with 5% non-fat dry milk. The primary antibodies used were a rabbit polyclonal antiserum raised against SERPING1 (IC1, 1/2500: Abcam, ab229209)and a mouse monoclonal antibody against albumin (HSA, diluted 1/1000: Abcam, ab10241). After washing, the membranes were incubated with a specific HRP-conjugated secondary antibody in PBS-T containing 5% non-fat dry milk and antibody binding was detected by enhanced chemiluminescence (ECL: GE Healthcare), according to the manufacturers’ instructions. Densitometry was performed with the ImageQuantTL software (GE Healthcare, IL, USA).

### 2.3. Functional Annotation Clustering

To examine the function of the proteins identified, the list of 33 proteins that varied significantly was analyzed with the on-line David Bioinformatics Resources 6.8 (NIH) software to assess their function [30]. Functional annotation clustering was performed to avoid any redundancy of enriched categories and pathways.

### 2.4. Statistical Analysis

Statistical analyses were performed using SPSS 15.0 for windows software (SPSS Inc., IL, USA). Continuous variables, such as age, were expressed as the mean ± standard deviation. After demonstrating the normal distribution of the population using a Kolmogorov–Smirnov test, a comparison of the means was performed using a Student T-test. Discrete variables, such as sex or the presence/absence of risk factors, were expressed as percentages. In these cases, a Fisher’s exact test was used to compare the groups. Statistical significance was accepted when *p*-value < 0.05.

For the TMT analysis, we considered differentially expressed proteins as those identified with at least two peptides and log2-ratios ˃│1.5│, expressed in the form of the standardized variables (Zq for proteins and Zp for the quantitation of oxCys peptide abundance) with *p*-values ≤ α < 0.05. To quantify peptide and protein abundance, changes were assessed with SanXoT software at a 1% FDR using the TMT reporter ion intensities from MS/MS scans as inputs to the weighted spectrum, peptide and protein (WSPP) model [31].

## 3. Results

In this work, we set out to study the effect of CAD on the calcification of the AV in CAVD using a high-throughput proteomic procedure based on the FASILOX technique. This study was carried out on a cohort of 20 patients with severe CAVD classified according to the absence or presence of CAD. By analyzing the proteome of the AV tissue obtained from these participants, we identified a total of 6353 peptides that corresponded to 1562 unique proteins in at least 90% of the samples. In addition, 570 peptides were identified that underwent reversible Cys oxidation.

**Figure 2:**
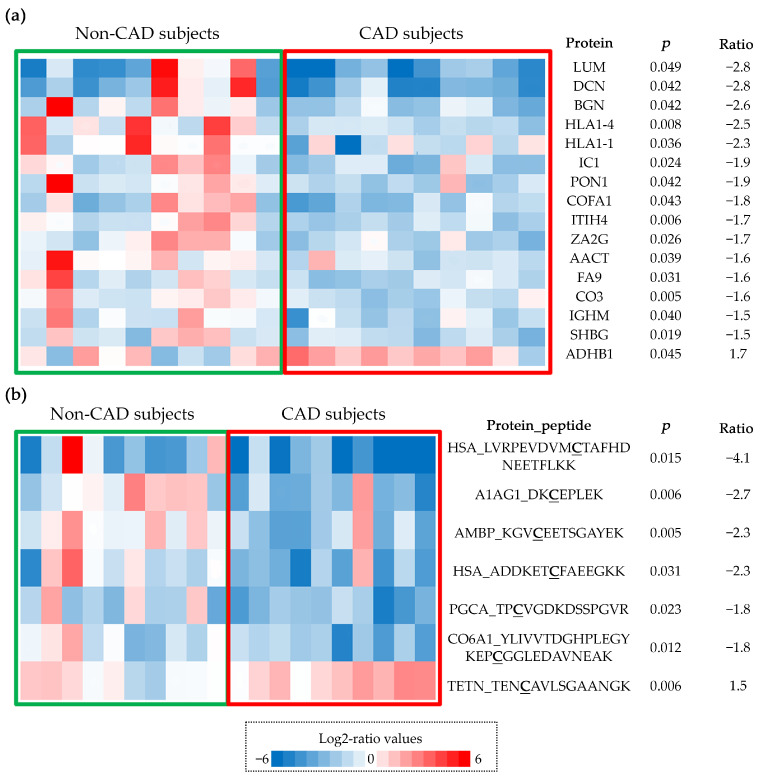
Proteins and oxidized peptides with differences in abundance between groups. Log2-ratio values are represented in a color scale (red means higher levels and blue lower levels); (**a**) heat-map representing abundance changes in differentially expressed proteins. Protein abbreviation, *p*-value and Zq log2-ratios are shown; (**b**) heat-map representing abundance changes in oxidized peptides. Protein abbreviation, measured peptide, *p*-value and Zp log2-ratios are shown. OxCys are bold and underlined. CAD, coronary artery disease.

After the statistical analysis, we identified 16 proteins that displayed differences in expression above 50% between the two study groups: lumican (LUM); decorin (DCN); collagen alpha-1(XV) chain (COFA1); biglycan (BGN); plasma protease C1 inhibitor (IC1); sex hormone-binding globulin (SHBG); inter-alpha-trypsin inhibitor heavy chain H4 (ITIH4); Ig mu chain C region (IGHM); zinc-alpha-2-glycoprotein (ZA2G); coagulation factor IX (FA9); HLA class II histocompatibility antigen, DRB1-4 beta chain (HLA-DRB1); serum paraoxonase/arylesterase 1 (PON1); complement C3 (CO3); HLA class II histocompatibility antigen, DRB1-1 beta chain (HLA-DRB1); alpha-1-antichymotrypsin (AACT); and alcohol dehydrogenase 1B (ADH1B). Except ADH1B, all these proteins were more strongly expressed in patients without CAD. It was notable that the heat-maps demonstrated a higher degree of homogeneity in the calcified AV tissue from patients with CAD than those from patients without CAD (Figure 2a), suggesting that the presence of CAD triggers specific events in the AV tissue, as discussed below.

In addition, seven oxCys peptides were seen to differ in their abundance between the two groups at an FDR of 1%. These peptides correspond to six proteins: protein AMBP (AMBP, Cys91); alpha-1-acid glycoprotein 1 (AGP, Cys183); collagen alpha-1(VI) chain (CO6, Cys169); human serum albumin (HSA, Cys148 and Cys591); aggrecan core protein (PGCA, Cys545); and tetranectin (TETN, Cys173). Except for the TETN peptides, all these peptides diminished in patients with CAD. According to Nexprot, these oxCys form disulfide bridges and, thus, they are important to maintain the 3D structure of the proteins. Interestingly, while oxidation of HSA has been studied extensively, modifications of Cys148 and Cys591 have yet to be described. Again, samples from patients with CAD were more homogeneous than the tissue from patients without CAD (Figure 2b).

### 3.1. Protein Functional Annotation

We performed a functional annotation analysis of the proteins differentially expressed between the two study groups, which resulted in three clusters (Table 3). Cluster 1 was formed by six proteins related to endopeptidase activity and platelet degranulation, with an enrichment score of 3.96. The second cluster was made up of five proteins related to the extracellular matrix (ECM), Golgi lumen and lysosomal lumen, with an enrichment score of 2.90. The finally cluster was formed by four proteins related to the proteinaceous ECM and its structural constituents, with an enrichment score of 2.43.

### 3.2. Verification Phase

We performed two different approaches to verify the results from the discovery phase in the plasma samples using orthogonal techniques to analyze independent cohorts of patients: PRM through MS analysis and immunodetection in Western blots. We focused on the proteins in cluster 1 because platelets play an important role in CAD, even exerting an effect on innate immunity, and this was the most intensely enriched and the largest cluster.

#### Targeted Proteomics (PRM) and Immunodetection (Western Blot)

**Figure 3:**
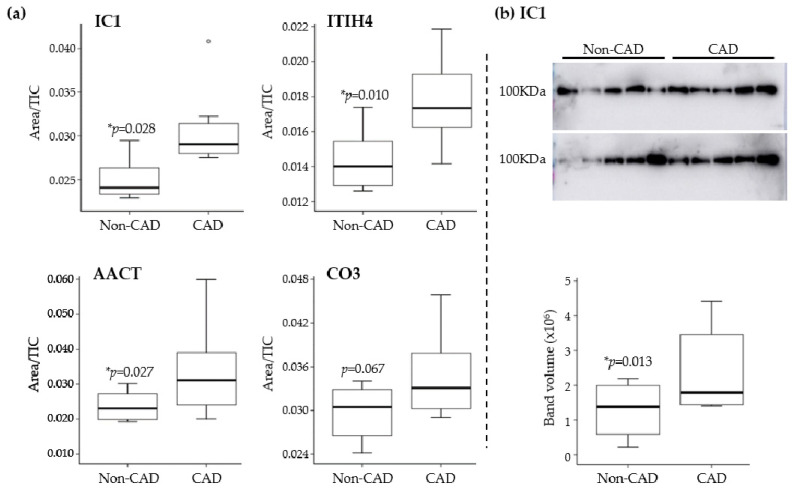
Results from plasma analysis in the verification phase. (**a**) Parallel reaction monitoringanalysis allow the measuring of plasma protease C1 inhibitor(IC1), inter-alpha-trypsin inhibitor heavy chain H4 (ITIH4), alpha-1-antichymotrypsin(AACT) and complement C3 (CO3), showing higher levels of protein in patients with coronary artery disease (CAD) in all cases. (**b**) Western blot of IC1, which confirmed the increased levels observed in parallel reaction monitoringanalyses. TIC, total ion chromatogram.

We first used PRM to quantify the proteins in cluster 1 and, of the six proteins, four were verified in the plasma samples from a different cohort of patients: ITIH4, IC1, AACT, and CO3. We measured 106 transitions corresponding to 16 peptides and we found higher levels of these proteins in the plasma from patients with CAD (Figure 3a). To validate these PRM results, we analyzed IC1, one of the proteins verified previously by this technique, using an orthogonal technique. The results from Western Blots confirmed the higher levels of this protein in CAD patients relative to the non-CAD patients (non-CAD = 1,332,081.1 ± 714,598.9 and CAD = 2,339,163.6 ± 1,111,596.5, *p*-value = 0.013: Figure 3b). 

### 3.3. Analysis of Cysteine Oxidation

**Figure 4:**
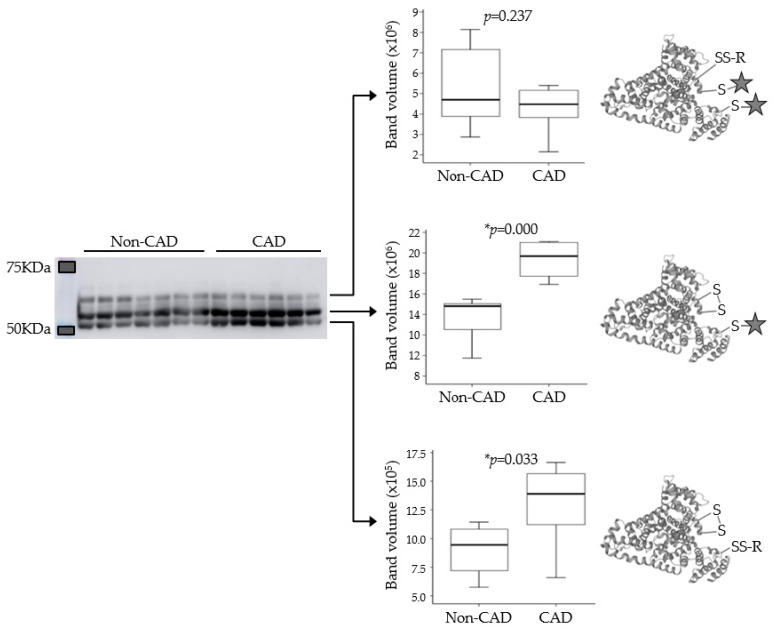
The thiol state of human serum albumin in patients with calcific aortic valve disease, with and without coronary artery disease (CAD). Western blot using -*SulfoBiotics*- labeling (star) showed that albumin with 1 and 2 oxCys were more abundant in patients with CAD. We did not find significant differences in the most reduced form of this protein.

Modification of protein thiols is one of the most important post-translational modifications and it occurs according to the redox state of cells. Differences in the oxidation of six peptides that corresponded to five proteins were found in the discovery phase, including two peptides of HSA with new sites of Cys oxidation. PEG-PCMal labeling allowed us to visualize changes in the redox state of this protein by electrophoretic analysis and immunodetection. In the case of HSA, we found three bands each separated by about 5 KDa (basal state, one oxCys and two oxCys: Figure 4), and an analysis of the immunoblot showed that the peptides with oneand twooxCys were more abundant in patients with CAD (basal state: non-CAD = 5,399,106.08 vs. CAD = 4,248,829.26, *p*-value = 0.237; 1 oxCys: non-CAD = 13,600,226.06 vs. CAD = 19,357,856.22, *p*-value = 0.000; 2 oxCys: non-CAD = 8,946,796.67 vs. CAD = 12,982,457.30, *p*-value = 0.033).

## 4. Discussion

In this study, we hypothesized that the presence of CAD may alter the course of AV calcification in CAVD at a molecular level. In addition, we wanted to see the difference in the redox state of the proteome of these valves. We found 16 proteins with different levels of expression between patients with severe CAVD with and without CAD. This number of differences is striking since the severe calcification of the AV is an effect associated with significant matrix disruption, irrespective of the presence of CAD. An analysis of the functional annotations identified three clusters of proteins in which the most abundant proteins were related to platelet degranulation and the regulation of endopeptidase activity, a cluster made up of six proteins: IGHM, PON1, ITIH4, IC1, AACT, CO3. We confirmed the relevance of ITIH4, IC1, AACT and CO3 in an independent cohort of plasma samples using orthogonal techniques. As such, this cluster highlighted the importance of platelets in both diseases [32,33,34,35], as well as their role in the intravascular innate immune system and in complement activation [36,37].

The differences in IC1 and CO3 highlight the importance of the innate immune system, indicating that anti-complement mediators are deposited in diseased AVs together with an activated complement [38]. Indeed, CO3 products and their cell receptors have been detected in atherosclerotic lesions of different severity in human arteries [39]. Nevertheless, the impact of the C3 complement system in the development of CAD is not clear and complement activation may exert dual atheroprotective and proatherogenic effects [40]. We also found alterations in ITIH4, an acute-phase response protein proposed to be a biomarker that serves to identify high-risk CAD patients [41]. Surprisingly, we found differences in AACT, a serine protease inhibitor that affects acute-phase proteins. There is evidence that this protein is released directly from the stenotic valve in response to the development of CAVD and that it is elevated in patients with CAVD [42], and we have now also seen differences in stenotic AVs associated with CAD. Despite the differences found in the expression of these proteins, the immune response is a non-specific mechanism and it is related to very different diseases, such as liver [43] and kidney [44] diseases or tumor progression [45]. Hence, the potential diagnostic value of these proteins could be less than that of proteins specific to CAD processes in patients with CAVD. Nevertheless, they should be taken into account as they could increase the sensitivity and specificity of diagnostic panels in combination with more specific proteins.

**Figure 5:**
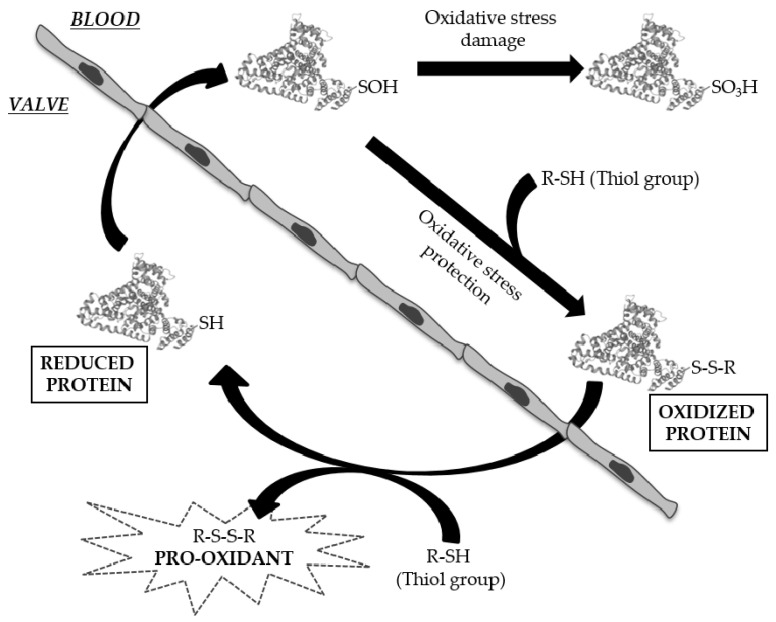
Possible biological significance of Cys modifications. Thiolation of circulating albumin (HSA) may confer protection against oxidative stress. These low-molecular-weight thiols may be released into the aortic valve and generate a pro-oxidant milieu.

One of the more exciting results presented here is the homogeneity among the valves of the group with CAD, as evident in the heat-maps. It appears that the presence of CAD triggers very specific processes in the AV that lead to its calcification. Differences in the development of CAD and CAVD have been widely studied [11,46], although it now seems clear that there are also different mechanisms that may lead to the calcification of AVs. These differences affect the redox state of the AV and we found fewer peptides with oxCys in the calcified AV tissue from patients with CAD, including two peptides from HSA. This protein has reactive Cys residues susceptible to some reversible or irreversible modifications, being protein S-thiolation by low-molecular-weight (LMW) thiols (cysteine-glycine (Cys-Gly), homocysteine (HCys), Cys, glutathione (GSH) and glutamylcysteine (Glu-Cys)), the most important reversible modification [47]. HSA contains 35 cysteine residues, 34 of which form disulfide bridges, and it has one free sulfhydryl group at position 34 (Cys34). The oxidative modification of this residue has been observed after infiltration in atherosclerotic plaques [48,49] and residue Cys34 plaque-filtered HSA was more oxidized than its corresponding circulating form, although we found more oxidized peptides in plasma samples. S-thiolation at Cys residues was recently described for the first time at the disulfide bonds of proteins in vivo, specifically at Cys90 and Cys101 of HSA [50]. Here, we have defined two new sites of oxidation, Cys148 and Cys591, which may fulfil functions distinct to Cys34, or they may even be oxidized specifically in patients with CAVD. We raise two hypotheses regarding the biological significance of these Cys modifications. Firstly, in its oxidized form, circulating HSA may act as a carrier of LMW thiols, which could be released into the subendothelial space to aggravate the oxidative stress suffered by the tissue (Figure 5). Secondly, the oxidation of Cys148 and Cys591 is likely to influence the tertiary structure of HSA and this may affect calcium binding, as it does in other molecules or drugs [51,52]. Nevertheless, these new sites of oxidation should be analyzed further as here we have only studied a limited number of subjects. Moreover, it will be interesting to perform functional studies to assess the true biological meaning of these oxidation events, as well as the role of the HSA in the development of CAVD. 

These results offer new insights into the role of HSA in the development of AV calcification in patients with CAD. Furthermore, the data presented here provide evidence of different molecular profiles in calcified AV depending on the presence or not of CAD, opening new avenues of research. Considering that CAVD is often associated with different co-morbidities, the possibility that AV calcification occurs through different molecular mechanisms lays the foundation for the study of new therapeutic strategies. This fact may also explain why there are controversial results regarding the use of some drugs to treat CAVD, such as statins [53,54,55,56]. If we focus on personalized medicine, defining the different mechanisms that affect AV in relation to each co-morbidity or risk factor is critical to offering adequate personalized treatment to each patient, improving the management of those who suffer from CAVD.

## 5. Conclusions

Here, we provide evidence that the presence of CAD in some way alters the proteome of calcified AVs, indicating that the development of CAVD is influenced by this co-morbidity. Indeed, the redox proteome is also altered in these circumstances and HSA seems to fulfil an important role in these events. Mass spectrometry experiments allowed us to define two new sites of Cys oxidation, perhaps representing a starting point for future functional studies. These results open the door to new studies into CAVD and its co-morbidities, and they may be of vital importance to take a step forward towards more personalized medicine for this condition.

## Figures and Tables

**Table 1:** Clinical characteristics of the subjects of study. CAVD, calcific aortic valve disease without coronary artery disease; CAVD + CAD, calcific aortic valve disease with coronary artery disease; M/F, male/female; *p*, *p*-value.

Clinical Characteristics	CAVD (*n* = 22)	CAVD + CAD (*n* = 22)	*p*
Age	75.9 ± 7.6	77.6 ± 6.5	0.435
Gender (%M/F)	60/40	32/68	0.073
%Obesity	5	14	0.300
%Hypertension	77	82	0.712
%Dyslipidemia	68	73	0.744
%Diabetes	32	45	0.359
%Smokers	18	9	0.385

**Table 2:** Targeted ions for parallel reaction monitoring analysis. Uniprot identifier of each protein, peptide sequence using the standard one-letter code for the amino acids, the mass-to-charge ratio (*m*/*z*), charge (z), start (Rt start) and stop (Rt stop) retention times (in minutes) are shown. AACT, alpha-1-antichymotrypsin; CO3, complement C3; IC1, plasma protease C1 inhibitor; ITIH4, inter-alpha-trypsin inhibitor heavy chain H4; min, minutes.

Protein	Peptide Sequence	*m*/*z*	Charge (z)	Rt Start (min)	Rt Stop (min)
P01011 AACT	DYNLNDILLQLGIEEAFTSK	1148.589	2	122.6	142.6
	DYNLNDILLQLGIEEAFTSK	766.0618	3	122.6	142.6
	EIGELYLPK	531.2975	2	66.2	86.2
	ITLLSALVETR	608.369	2	92	112
	NLAVSQVVHK	547.8195	2	37	57
	NLAVSQVVHK	365.5487	3	36.8	56.8
P01024 CO3	EYVLPSFEVIVEPTEK	939.9904	2	96.2	116.2
	EYVLPSFEVIVEPTEK	626.996	3	96.2	116.2
	VEGTAFVIFGIQDGEQR	933.4732	2	90	110
	VEGTAFVIFGIQDGEQR	622.6513	3	90	110
	VHQYFNVELIQPGAVK	921.4991	2	75.8	95.8
	VHQYFNVELIQPGAVK	614.6685	3	75.8	95.8
	VPVAVQGEDTVQSLTQGDGVAK	1099.569	2	67.8	87.8
	VPVAVQGEDTVQSLTQGDGVAK	733.3815	3	67.8	87.8
P05155 IC1	GVTSVSQIFHSPDLAIR	913.9916	2	80.2	100.2
	GVTSVSQIFHSPDLAIR	609.6635	3	80.2	100.2
	LEDMEQALSPSVFK	797.3951	2	78.9	98.9
	LLDSLPSDTR	558.7984	2	48.7	68.7
	TNLESILSYPK	632.8428	2	77.7	97.7
Q14624 ITIH4	GPDVLTATVSGK	572.8141	2	46.9	66.9
	LGVYELLLK	524.3261	2	88.7	108.7
	NGIDIYSLTVDSR	726.8701	2	77	97
	TGLLLLSDPDK	586.3321	2	73.3	93.3

**Table 3:** Functional analysis of the differentially expressed proteins. The proteins are represented in clusters according to their function. The enrichment score (enrich. score), number of terms (N. of terms) and proteins included in each cluster are shown. IGHM, Ig mu chain C region;PON1, serum paraoxonase/arylesterase 1; ITIH4, inter-alpha-trypsin inhibitor heavy chain H4; IC1, plasma protease C1 inhibitor; AACT, alpha-1-antichymotrypsin; CO3, complement C3; BGN, biglycan; DCN, decorin; COFA1, collagen alpha-1(XV) chain; LUM, lumican; FA9, coagulation factor IX.

Cluster	Function	Enrich. Score	N. of Terms	Proteins
1	Platelet degranulation and regulation of endopeptidase activity	3.96	4	IGHM, PON1, ITIH4, IC1, AACT, CO3
2	Extracellular matrix; Golgi and lysosomal lumen	2.90	4	BGN, DCN, COFA1, LUM, FA9
3	Extracellular matrix (structural)	2.43	3	BGN, DCN, COFA1, LUM

## Data Availability

Data is contained within the article and supplementary material.
